# A cross-sectional survey of adrenal steroid hormones among overweight/obese boys according to puberty stage

**DOI:** 10.1186/s12887-019-1755-5

**Published:** 2019-11-06

**Authors:** Bingyan Cao, Chunxiu Gong, Di Wu, Xuejun Liang, Wenjing Li, Min Liu, Chang Su, Miao Qin, Xi Meng, Liya Wei

**Affiliations:** Department of Endocrinology, Genetics and Metabolism, Beijing Children’s Hospital, Capital Medical University, National Center for Children’s Health, 56#Nanlishi Road, West District, Beijing, 100045 China

**Keywords:** Obesity, Adolescent boys, Adrenal steroid hormones

## Abstract

**Background:**

Obesity is associated with many chronic diseases including cortisol rhythm disorder and low testosterone. Furthermore, studies on obese children are quite limited and no concordance results have been obtained, especially for boys in puberty. Moreover, the sample sizes of previous studies were small, and were not representative.

**Methods:**

We conducted a cross-sectional survey including 1148 boys aged 6–14 years, they were divided into overweight/obesity (OW/OB) group and normal weight (NW) group. Puberty status was assessed according to Tanner scale and testicular volume. Serum levels of pregnenolone, 17-OH progesterone, corticosterone, dehydroepiandrosterone (DHEA), and androstenedione were detected by LC-MS. Serum free testosterone and sex hormone-binding globulin (SHBG) levels were measured by chemiluminescence immunoassay.

**Results:**

The 17-OH progesterone, DHEA, androstenedione and free testosterone levels of OW/OB boys at prepubertal stage or at the age 6 = < 10 years group were higher than those of the NW boys (all the *P* values were < 0.01). Furthermore, androstenedione and free testosterone levels were lower in OW/OB boys at late puberty, and the trend continued at the post pubertal stage for FT (*P* < 0.01–0.05). DHEA, androstenedione, and FT levels persisted to be higher at the 10~ < 12 years in OW/OB boys but not for 17-OH progesterone. FT level was lower in the OW/OB group at the 12~ < 15 years group. The SHBG levels in the OW/OB boys were lower than those in the NW ones at the 6~12 years group, and prepubertal to early pubertal stage.

**Conclusions:**

Premature adrenarche is more likely in OW/OB boys. More attention should be given to the lower androgen levels of OW/OB boys at late pubertal and post pubertal stages.

## Background

Obesity is associated with many chronic disorders such as anxiety, depression, high blood pressure, hyperlipidemia, type 2 diabetes mellitus, obstructive sleep apnea, certain types of cancer, osteoarthritis, and asthma [1–3]. Lower testosterone levels in obese adult males increase the risk of metabolic diseases. In addition, relatively higher levels of cortisol increase the risk of metabolic diseases in OB children [4–6]. Children and adolescents with obesity have a higher incidence of polycystic ovary syndrome and precocious puberty (these apply mainly to females), and tall stature associated with advanced bone maturation [7, 8]. Dehydroepiandrosterone (DHEA) may be involved in the regulation of the growth rate of bone age [9], in which abnormal increase may accelerate the growth of body height to a certain extent. This raises the question that obesity may have different influence on androgen hormones among different pubertal stages and different age boys.

Limited studies have been performed on steroid hormones in OB children, and no concordance results have been obtained to date, studies on steroid levels in OB children have shown that TT levels in prepubertal OB boys were higher than [10, 11], or lower [12] than those in children with normal weight (NW) [13]. In addition, in pubertal OB boys, TT level has been reported to be normal [10, 14] or decreased [15, 16]. Indeed, some defective factors exist in those studies such as the observed sample numbers were small, there was no strict evaluation of puberty, and some studies used immunological methods which might have affected the reliability of the results. These might have caused the different results. The result from the study on OB children showed free testosterone (FT) may be a better observable index than TT in reflecting gonadal function [17]. Limited studies concerned about FT in obese children [14, 15, 17], and showed FT was higher than normal children before pubertal onset and at early pubertal stage, similar FT at mid and late puberty stage, lower at post pubertal stage than normal children [17].The only study conducted on glucocorticoid and mineralocorticoid in adolescent OB children revealed that 11-deoxycortisol, cortisol and corticosterone levels were elevated, but declined once body weight was reduced [11]. These results suggested obesity in childhood could result in elevated steroid hormones (i.e. cortical hyperfunction). Therefore, in the present study, six adrenal cortex steroid hormones (pregnenolone, 17-OH progesterone, corticosterone, DHEA, and FT) of three main zones and sex hormone-binding globulin (SHBG) were analyzed, and genital development was assessed on 6–14 year-old school boys in a cross-sectional survey. We hypothesized that obese prepubertal boys have higher androgen hormones (FT, DHEA, androstenedione), late pubertal boys have lower androgen hormones concentrations as compared to lean boys.

## Methods

### Enrolled boys

It was a cross-sectional survey conducted in Shunyi District, Beijing, 3 elementary schools and 3 middle schools were selected by the cluster sampling method. A total of 1220 Chinese boys aged 6 to 14 years took part in the study, and 72 boys (5.90%) with history of hypogonadism, primary hyperlipidemia, hypertension, diabetes mellitus, impaired fasting glucose, impaired glucose tolerance, syndromic or endocrine obesity, or those undergoing pharmacological treatment were excluded. At last, total 1148 (94.10%) boys completed the survey. The heights, weights, waist circumferences (WC) and hip circumferences of male students were recorded. The puberty staging was performed using the Tanner scale (refer to the methods below). This study was authorized by the Ethics Committee of Beijing Children’s Hospital affiliated to Capital Medical University (Beijing, China). All parents of the enrolled children in this study were informed and provided a signed a consent to provide the data for this study. All the boys were divided into three age groups, 6~ < 10 years, 10~ < 12 years, 12~ < 15 years.

### Puberty stage assessment

The pubertal stage assignment and testicular volume estimation were performed by well-trained pediatric endocrinology specialist using a Prader orchidometer. Subjects were classified according to their testicular volume and pubic hair for Tanner stages. The puberty stages were assessed as follows: boys with testicular volume of 3 ml or less were considered prepuberty, 4~8 ml were considered early puberty, > 8-15 ml were considered mid puberty, > 15-20 ml were considered late puberty, 20–25 ml were considered post puberty. Pubic hair stages (PH1–PH5) were recorded according to Tanner [18].

### Group classification

Students were divided into two groups, according to their body mass index (BMI): OW/OB group, and NW group [19]. BMI was calculated by dividing the weight (kg) from the square of the height (m). The diagnostic criteria for OW and OB was based on the Chinese children BMI percentile table, in which OW represents a BMI level that exceeds the 85–95 percentile of the same age boy and OB represents a BMI level that exceeds > 95 percentiles of the same age boy [19].

### Biochemical assessment

The serum samples were acquired using a protocol as described previously for determining the concentrations of steroid hormones [20]. The LC-MS method was used to assess pregnenolone, 17α hydroxyprogesterone, corticosterone, DHEA, and androstenedione levels, FT and SHBG levels were measured using Chemiluminescence Immunoassays (CLIA) assay, both of the above biochemical assessment methods were similar to those described before [20].

### Statistics analysis

The SAS JMP (SAS Institute INC. Cary, NC) was used for data analyses. Normality was checked using Shapiro-Wilk tests. The anthropometric data and hormonal data were not normally distributed. Non-normal distribution data were expressed as (25th–75th percentiles). Differences between groups were determined using the Wilcoxon’s non-parametric test. Hormonal parameters pregnenolone, 17-OH progesterone, corticosterone, DHEA, androstenedione, SHBG and FT underwent a logarithmic transformation to enhance normality, linear regression analysis was used to test the effect of BMI-SDS, WC-height ratio on the above steroid hormones in prepubertal boys and those having a testis volume > 3 ml together. *P* < 0.05 was considered statistically significant.

## Results

### General information

The general information of the enrolled 1148 boys is listed in Table 1. There were 353 OW/OB boys, accounted for 30.56% of all the enrolled boys. Among them, 620 boys were in prebuberty stage with 25% OW/OB rate, 216 boys were in early puberty stage with 46.30% OW/OB rate, 106 boys were in mid puberty stage with 33.96% OW/OB rate, 145 boys were in late puberty stage with 30.34% OW/OB rate, and 61 boys were in post puberty stage with 29.51% OW/OB rate (Table 1). Interestingly, the OW/OB rate was lowest in the prepuberty, closed to which in post puberty, highest in early puberty, and relatively higher in mid puberty and late puberty.

**Table 1:** Comparison of anthropometric data in different pubertal stages between the NW and OW/OB groups

Group		NW	OW/OB	Z value	*p*
Prepuberty	n	465	155		
	Age (year)	8.27 (6.92, 9.54)	9.21 (7.93, 10.16)	5.47	< 0.0001
	Weight (kg)	26.1 (23.0, 30.0)	43.0 (38.50, 51.0)	17.17	< 0.0001
	BMI-SDS	−0.23 (− 0.77,0.33)	3.29 (2.33, 4,77)	18.92	< 0.0001
	WC/height	0.42 (0.40–0.44)	0.54 (0.50–0.57)	17.72	< 0.0001
Early puberty	*n*	116	100		
	Age (year)	11.44 (10.47, 12.36)	11.12 (10.47, 11.78)	−2.62	0.01
	Weight (kg)	36.00 (33.63, 40.00)	52.45 (47.20, 59.88)	11.77	< 0.0001
	BMI-SDS	−0.18 (−0.72, 0.33)	2.69 (2.010, 3.96)	12.92	< 0.0001
	WC/height	0.41 (0.40–0.43)	0.52 (0.50–0.56)	12.18	< 0.0001
Mid puberty	*n*	70	36		
	Age (year)	13.42 (12.50, 14.12)	12.70 (11.62, 13.49)	−3.06	0.003
	Weight (kg)	45.00 (41.50, 50.00)	60.70 (53.30, 76.13)	6.66	< 0.0001
	BMI-SDS	−0.17 (−0.60, 0.55)	2.70 (1.97, 4.18)	8.42	< 0.0001
	WC/height	0.40 (0.39–0.42)	0.51 (0.49–0.56)	9.92	< 0.0001
Late puberty	*n*	101	44		
	Age (year)	13.99 (13.38, 14.40)	13.01 (11.82, 13.98)	−3.98	< 0.0001
	Weight (kg)	50.00 (46.75, 54.50)	64.50 (54.63, 76.25)	6.7	< 0.0001
	BMI-SDS	−0.09 (−0.51, 0.42)	2.69 (1.85, 3.50)	9.55	< 0.0001
	WC/height	0.41 (0.39–0.42)	0.51 (0.46–0.53)	6.60	< 0.0001
Post puberty	*n*	43	18		
	Age (year)	14.26 (14.00, 14.66)	13.84 (13.24, 14.23)	2.78	0.005
	Weight (kg)	56.00 (51.50, 61.00)	81.00 (69.50, 93.25)	5.54	< 0.0001
	BMI-SDS	−0.30 (−0.31, 0.77)	3.52 (2.60, 5.32)	6.11	< 0.0001
	WC/height	0.41 (0.39–0.43)	0.51 (0.46–0.56)	5.31	< 0.0001

Values are presented as median and interquartile range (IQR)

*NW* normal weight, *OW/OB* overweight/obese, *WC* waist circumference

### Adrenal steroid hormone levels in different pubertal stages

The serum levels of pregnenolone, corticosterone, 17-OH progesterone, DHEA, androstenedione, and FT in the NW and OW/OB groups at different pubertal stages were measured, and the results are listed in Table 2. These results revealed that pregnenolone and corticosterone levels in the NW group did not show a significant alteration during pubertal development. On the other hand, the serum levels of 17-OH progesterone, DHEA, androstenedione and FT in the NW group presented a gradually increasing tendency from the prepuberty to the post puberty stage. After adjustment for age difference, each hormone level in the OW/OB groups exhibited dissimilar alterations at different stages of adolescence, compared to the corresponding NW boys. Serum pregnenolone levels in the OW/OB groups at the prepuberty, mid puberty to post puberty phases did not reveal a significant difference, at the early puberty phase which presented a significant decrease, compared to the corresponding NW groups (0.09 vs. 0.13 μg/L, *P* < 0.01). The 17-OH progesterone levels in the OW/OB group at the early puberty to post puberty phases were not different compared to the NW groups. However, the level at the prepuberty phase was notably higher than that in the NW group (0.34 vs.0.22 ng/mL, *P* < 0.01).The DHEA, androstenedione, and FT levels in the OW/OB group were significantly higher than in the NW group at the prepuberty phase (1.49 vs.0.70 g/L, *P* < 0.01; 0.24 vs. 0.18 g/L, *P* < 0.01; 1.26 vs.0.97 pg/mL, *P* < 0.01, respectively). The androstenedione and FT levels in the OW/OB group were lower than those in the NW group at the late puberty phase (0.63 vs. 0.53 μg/L, *P* < 0.05 and 15.76 vs. 26.39 pg/mL, *P* < 0.01, respectively). The FT levels of the OW/OB boys were lower than those in the NW group, as well as in the post puberty phase (24.47 vs. 34.16 pg/mL, *P* < 0.05). The corticosterone levels of OW/OB boys did not show any significant alteration compared to those in the NW group at all pubertal stages. Serum SHBG levels were measured in 344 boys, which showed a gradual decline along with the development of puberty in NW boys. Due to the limited sample size, the SHBG levels between the OW/OB and NW groups at the prepuberty, early puberty and mid puberty phases were compared, which revealed that the SHBG of OW/OB boys were obviously lower than those in the NW group (prepuberty: 112.7 vs. 64.5 nmol/L, *P* < 0.01 and early puberty: 79.54 vs. 45.1 nmol/L, *P* < 0.01, respectively).

**Table 2:** Serum adrenal steroid levels in both the NW and OW/OB groups at different pubertal stages

	Tanner stage	NW		OW/OB		*P*
		N	Value	*N*	Value	
Pregnenolone (ug/l)	Prepuberty	383	0.08(0.05–0.13)	141	0.08(0.05–0.12)	0.24
	**Early puberty**	**97**	**0.13(0.09–0.21)**	**93**	**0.09(0.05–0.14)**	**< 0.0001**
	Mid puberty	67	0.16(0.11–0.25)	31	0.12(0.06–0.21)	0.14
	Late puberty	94	0.18(0.13–0.23)	36	0.19(0.11–0.29)	0.78
	Post puberty	39	0.16(0.12–0.21)	18	0.20(0.11–0.29)	0.48
Corticosterone(ug/l)	Prepuberty	465	1.45 (0.87–2.92)	155	1.41(0.77–3.00)	0.59
	Early puberty	116	1.36 (0.80–2.98)	100	1.62(0.88–3.38)	0.32
	Mid puberty	70	1.38(0.63–2.54)	36	1.93(0.85–2.80)	0.21
	Late puberty	101	1.31 (0.75–2.25)	44	1.07(0.72–2.80)	0.49
	Post puberty	43	1.58 (0.74–2.93)	18	1.96(0.88–4.21)	0.30
17OH- progesterone (ng/ml)	**Prepuberty**	**465**	**0.22 (0.15–0.38)**	**155**	**0.34(0.22–0.49)**	**< 0.0001**
	Early puberty	116	0.37 (0.26–0.51)	100	0.40(0.33–0.58)	0.05
	Mid puberty	70	0.53 (0.36–0.75)	36	0.54(0.43–0.66)	0.59
	Late puberty	101	0.65 (0.47–0.86)	44	0.51(0.39–0.84)	0.06
	Post puberty	43	0.68 (0.49–0.94)	18	0.62(0.54–0.88)	0.47
DHEA (ug/l)	**Prepuberty**	**465**	**0.70 (0.37–1.34)**	**155**	**1.49(0.88–2.31)**	**< 0.0001**
	Early puberty	116	2.12 (1.56–3.16)	100	2.31(1.66–3.30)	0.23
	Mid puberty	70	3.10 (2.21–4.24)	36	3.34(2.26–5.07)	0.34
	Late puberty	101	3.65 (2.64–4.59)	44	3.26(2.20–4.32)	0.41
	Post puberty	43	4.36 (2.83–5.49)	18	3.57(2.54–5.55)	0.98
Androstenedione (ug/l)	**Prepuberty**	**465**	**0.18 (0.13–0.25)**	**155**	**0.24(0.17–0.33)**	**< 0.0001**
	Early puberty	116	0.32 (0.23–0.44)	100	0.36(0.27–0.44)	0.37
	Mid puberty	70	0.48 (0.34–0.65)	36	0.52(0.30–0.72)	0.86
	**Late puberty**	**101**	**0.63 (0.48–0.77)**	**44**	**0.53(0.40–0.68)**	**0.02**
	Post puberty	43	0.71 (0.57–0.90)	18	0.67(0.56–0.82)	0.85
FT (pg/ml)	**Prepuberty**	**465**	**0.97 (0.59–1.36)**	**155**	**1.26(0.90–1.76)**	**< 0.0001**
	Early puberty	116	2.24 (1.69–3.49)	100	2.34(1.61–3.11)	0.68
	Mid puberty	70	11.52 (5.66–23.35)	36	6.79 (3.52–19.18)	0.09
	**Late puberty**	**101**	**26.39 (17.12–37.36)**	**44**	**15.76 (8.52–26.07)**	**0.0006**
	**Post puberty**	**43**	**34.16 (24.98–47.07)**	**18**	**24.47 (17.90–32.00)**	**0.02**
SHBG (nmol/L)	**Prepuberty**	**129**	**112.7 (87.8–150.2)**	**29**	**64.5 (41.6–117.1)**	**< 0.0001**
	**Early puberty**	**42**	**79.54 (65.75–110.75)**	**31**	**45.1 (34–71.2)**	**< 0.0001**
	Mid puberty	24	43.25 (29.95–58.15)	11	35 (17–50.8)	0.12

Values are presented as median and interquartile range (IQR)

SHBG levels between the OW/OB and NW groups at the prepuberty, early puberty and mid puberty phases were compared. SHBG levels at the late puberty and post puberty phases between the OW/OB and NW groups were not compared due to the limited sample sizes

*NW* normal weight, *OW/OB* overweight/obese, *FT* Free testosterone, *DHEA* Dehydroepiandrosterone, *SHBG* Sex hormone-binding globulin

The comparisons of steroid hormones between OW/OB groups and NW groups in different pubic hair staging were shown in Additional file 1: Table S1, we combined Tanner stage 2 and 3 for eliminating the inter-observer variation, Tanner stage 4 and 5 were combined for analyses because of the limited sample sizes.

### Steroid hormone levels in different age groups (results shown in Table 3)

The serum levels of 17-OH progesterone, DHEA, androstenedione, and FT in the OW/OB groups were higher than those in the NW groups at the 6~ < 10 years group, DHEA, androstenedione, and FT persisted higher at the 10~ < 12 years but not for 17-OH progesterone. However, the pregnenolone level in OW/OB group was lower than in NW group at the same age group. FT showed lower in the OW/OB group at the 12~ < 15 years group. The SHBG level in the OW/OB group was lower in the NW group at the both 6~ < 10 years and 10~ < 12 years group.

**Table 3:** Serum adrenal steroid levels in both the NW and OW/OB groups at different age groups

	Age group	NW		OW/OB		*P*
		N	Value	*N*	Value	
Pregnenolone (ug/l)	6- < 10 years	370	0.08(0.05–0.12)	111	0.07(0.04–0.11)	0.29
	**10- < 12 years**	**120**	**0.11(0.07–0.18)**	**135**	**0.08(0.05–0.13)**	**0.001**
	12- < 15 years	218	0.17(0.12–0.23)	73	0.18(0.11–0.29)	0.42
Corticosterone(ug/l)	6- < 10 years	400	1.45 (0.88–2.86)	117	1.47(0.96–3.36)	0.45
	10- < 12 years	162	1.44 (0.83–2.98)	148	1.48(0.78–3.11)	0.85
	12- < 15 years	234	1.33(0.74–2.76)	87	1.50(0.77–2.98)	0.49
17OH-progesterone (ng/ml)	**6- < 10 years**	**400**	**0.20 (0.14–0.31)**	**117**	**0.32(0.19–0.49)**	**< 0.001**
	10- < 12 years	162	0.38 (0.26–0.58)	148	0.41(0.31–0.57)	0.11
	12- < 15 years	234	0.57 (0.42–0.81)	87	0.56(0.42–0.79)	0.77
DHEA (ug/l)	**6- < 10 years**	**400**	**0.57 (0.28–0.96)**	**117**	**1.42(0.80–2.11)**	**< 0.0001**
	**10- < 12 years**	**162**	**1.85 (1.26–2.73)**	**148**	**2.27(1.58–3.27)**	**0.004**
	12- < 15 years	234	3.40 (2.48–4.48)	87	3.51(2.46–5.12)	0.24
Androstenedione (ug/l)	**6- < 10 years**	**400**	**0.14 (0.10–0.20)**	**117**	**0.23(0.15–0.32)**	**< 0.0001**
	**10- < 12 years**	**162**	**0.30 (0.21–0.42)**	**148**	**0.36(0.27–0.45)**	**0.0035**
	12- < 15 years	234	0.58 (0.40–0.76)	87	0.56(0.40–0.73)	0.51
FT (pg/ml)	**6- < 10 years**	**400**	**0.87 (0.52–1.22)**	**117**	**1.21(0.85–1.69)**	**< 0.0001**
	**10- < 12 years**	**162**	**1.82 (1.42–2.85)**	**148**	**2.21(1.56–3.37)**	**0.02**
	**12- < 15 years**	**234**	**19.78 (8.22–34.40)**	**87**	**15.26(4.64–25.16)**	**0.01**
SHBG (nmol/L)	**6- < 10 years**	**96**	**110.9 (86.45–146.15)**	**25**	**71.1 (45.75–121.75)**	**0.002**
	**10- < 12 years**	**64**	**102.4 (70.80–157.10)**	**33**	**45.10 (32.55–71.00)**	**< 0.0001**
	12- < 15 years	103	36.50 (28.00–49.30)	23	36.90 (19.20–55.70)	0.56

Values are presented as median and interquartile range (IQR)

*NW* normal weight, *OW/OB* overweight/obese, *FT* Free testosterone, *DHEA* Dehydroepiandrosterone. SHBG: Sex hormone-binding globulin

### Associations between the degree of obesity and steroid hormone levels

In prepubertal boys, BMI-SDS was a positive predictor of 17-OH progesterone (BMI SDS, β = 0.07; *P* < 0 .01), DHEA (BMI SDS, β = 0.12; *P* < 0.01), androstenedione (BMI SDS, β = 0.06; *P* < 0 .01) and FT (BMI SDS, β = 0.04; *P* < 0 .01) levels, however, BMI SDS was a negative predictor of SHBG (BMI SDS, β = − 0.07, *P* < 0.01) level. In all boys with testis volume > 3 ml, BMI-SDS was a negative predictor of pregnenolone (BMI SDS, β = − 0.07; *P* < 0 .01), FT (BMI SDS, β = − 0.11; *P* < 0 .01), SHBG (BMI SDS, β = − 0.05, *P* < 0.01) levels. However, no association between BMI SDS and 17-OH progesterone, DHEA, androstenedione was found. All results remained unchanged when BMI SDS was substituted by WC to height ratio in the analyses (data were not shown).

## Discussion

Understanding the alteration of adrenal steroid hormone levels in different adolescent stages of OB boys would be helpful for accurate diagnosis and treatment. In the present study, we investigated the serum levels of intermediate products of the adrenal glomerulosa zone (corticosterone), fasciculate zone (17-OH progesterone), and reticular zone hormones (DHEA, androstenedione and FT) by comparing 6–14 year-old OW/OB boys to NW ones. These results revealed that the serum pregnenolone and corticosterone levels of NW boys did not reveal a significant change during pubertal development. However, 17-OH progesterone, DHEA, androstenedione and FT progressively increased with puberty development. The serum levels of 17-OH progesterone from fasciculate zone, DHEA, androstenedione and FT from the reticular zone in OW/OB boys were higher than those in NW boys at prepuberty or aged 6~10 years, in which the alteration was closely correlated with body mass index and WC/height. The result of increasing FT levels in OW/OB boys at prepuberty stage is consistent with the findings of Vandewalle and Reinehr [11, 17]. It is a relatively consistent result that concerns FT levels in prepubertal OB boys from the available studies. In addition to FT, the present results and data from the study conducted by Vandewalle et al. and Reinehr etal [11, 14] revealed that DHEA and androstenedione significantly elevated at the prepuberty stage in OW/OB boys. The increased FT may result from increased adrenal activity as revealed by elevated serum DHEA [21], which suggests that the premature adrenarche is more likely in OW/OB boys. The increase in production of adrenal testosterone precursors may correlate with an increased central activation of the HPA axis, CRH has been suggested to increase adrenal androgen secretion directly in children [21]. The increased insulin concentrations in obese prepubertal children could activate important enzymatic step for androgen formation, Δ5-Δ4 conversion of dehydroepiandrosterone to androstenedione, 3β-hydroxysteroid dehydrogenase expression can be enhanced via an insulin receptor substrate-1- and − 2-dependent pathway in different tissue types [22]. In addition, leptin secreted by the adipose tissue may also act at the level of adrenal steroid biosynthesis, it has been verified to promote the formation of adrenal androgens by stimulating 17,20-lyase activity of CYP17 [23]. All of these factors might attribute to the increased androgens in the adrenals and in the periphery among prepubertal OW/OB boys.

17-OH progesterone is an intermediate product from the corticosteroid cortisol synthetic process. Our data revealed that the serum level of 17-OH progesterone in OW/OB boys increased at prepuberty and aged 6~10 years. However, Reinehr et al. [11] reported that there was no significant difference in 17-OH progesterone level between 40 prepubertal OB boys and 40 weight-matched and puberty stage-matched normal children. We believe that in their study, the number of boys observed was very small to obtain such definitive result.

In the present study, corticosterone, mineralocorticoid precursors, dose not exhibit an obvious change. In addition, Reinehr et al. [11] found that corticosterone levels elevated in OB boys, while the true precursor substance of the mineralocorticoid, 11-deoxycorticosterone, did not change. After weight loss, corticosterone decreased, but 11-deoxycorticosterone and aldosterone did not change; indicating that obesity had no effect on the glomerulosa zone hormone. These results suggest that obesity does not affect corticosterone production either before or after puberty.

Furthermore, in the present study, we found that serum FT level was lower in OW/OB boys than NW boys at the late puberty and post puberty stage, which was consistent with the results of Vandewalle (in mature obese adolescents) and Mogri (in obese late pubertal and post pubertal adolescents) [14, 15]. Androstenedione level was also lower in OW/OB boys than NW boys at the late puberty stage. There are some changes among OB boys may help to explain it. Hypogonadotropic hypogonadism may occur in severe obesity with a significantly decreased luteinizing hormone (LH) secretion peak [24, 25]. Reinehr et al. reported that testosterone levels increased after weight loss in OB boys [11]. This testosterone level increase may be caused by LH secretion after weight loss in OB boys [26]. Thus, these data suggests that the lower sex hormone levels of OB children may be affected by the decreased activity of the HPG axis at adolescence. It is known that insulin and leptin stimulate the expression of hypothalamic GnRH [27, 28], obesity correlated with insulin resistance and leptin resistance, both of these two factors may result in subnormal secretion of GnRH at the neuronal level. It also has been documented that obesity-induced metabolic inflammation affects the function of HPG axis [29]. The above factors maybe the potential mechanism linking male obesity with HPG axis defects at the late puberty and post puberty stage.

The present study revealed that BMI was negatively related to FT among boys having testis volume > 3 ml. Previous studies have concluded that BMI, waist circumference and estrogen levels were the negative feedback regulation by LH, and the greater the degree of obesity, the more obvious the negative feedback regulation became [30, 31]. In OB adult men, low testosterone reduces lean body mass, muscle capacity and strength; and decreases bone mineral density. It increases the risks of type 2 diabetes, dyslipidemia, obesity, hypertension, cardiovascular disease and death. Low testosterone can cause a decline in sexual desire, erectile dysfunction, decrease in ejaculation function, fatigue, depression, and decline in cognition and intellect. In addition, the supplementation of testosterone in males has been believed to be a safe and effective to ameliorate these conditions [32, 33]. Nonetheless, the relationship between the low levels of testosterone in obese children and these disorders remains to be confirmed, further studies also needed to clarify the necessity, safety and effectiveness of the supplementation of testosterone in late adolescent children.

## Conclusions

In conclusion, our study revealed that the adrenal androgens DHEA and androstenedione are increased in prepubertal obese vs NW boys, consistent with the known increase in the frequency of premature adrenarche in obese children. In addition, more attention should be given to the lower androgen levels of OW/OB boys at late pubertal and post pubertal stages. The characteristics of the present study are as follows: (1) The study population was a cross-sectional survey of primary and secondary school students, which is different from the previous study that focused on hospital-visiting populations or participants in a weight losing program of OB children. Our study represents the true distribution of the normal and general populations. (2) More population was enrolled into the present study, especially for pre-adolescent and early adolescent children. (3) Most of the hormones were analyzed using a sensitive and specific method, LC-MS, except for FT and SHBG. (4) This study is the first to explore the alteration of adrenal glucocorticoid and mineralocorticoid precursors in OB children at different stages of adolescence. (5) The adolescent stages of the boys were classified more scientifically. On the other hand, the weak points of this study were as follows: (1) The serum cortisol and aldosterone levels were not detected in this study. (2) The sample size of children at adolescent maturity is not enough.

## Supplementary information

**Additional file 1: Table S1.** Serum adrenal steroid levels in both the NW and OW/OB groups at different Tanner genital stages.

## Data Availability

The datasets used and/or analysed during the current study are available from the corresponding author on reasonable request.

## Abbreviations

BMI: Body mass index
CLIA: Chemiluminescence Immunoassays
CRH: Corticotropin-releasing hormone
CYP17: 17 α hydroxylase
DA: Androstenedione
DHEA: Dehydroepiandrosterone
FT: Free testosterone
GnRH: Gonadotropin releasing hormone
HPA: Hypothalamus-pituitary-adrenal
HPG: Hypothalamus-pituitary-gonadal
LC-MS: Liquid chromatography–mass spectrometry
NW: Normal weight
OW/OB: Over-weight/obesity
SHBG: Sex hormone-binding globulin
TT: Total testosterone
WHO: World Health Organization
