# Supplementary material for: A cross-sectional survey of adrenal steroid hormones among overweight/obese boys according to puberty stage
Source: BMC Pediatr. 2019 Nov 6;19:414. doi: 10.1186/s12887-019-1755-5 (PMC6833276; doi:10.1186/s12887-019-1755-5)
Supplement: Supplementary file 1 — Additional file 1: Table S1. Serum adrenal steroid levels in both the NW and OW/OB groups at different Tanner genital stages. [file 12887_2019_1755_MOESM1_ESM.docx]

Additional file 1: Table S1. Serum adrenal steroid levels in both the NW and OW/OB groups at different Tanner genital stages

|  | Puberty stage | NW |  | OW/OB |  | *P* |
| --- | --- | --- | --- | --- | --- | --- |
|  |  | N | Value | *N* | value |  |
| Pregnenolone  (ug/l) | G1 | 510 | 0.08 (0.05-0.14) | 241 | 0.08 (0.05-0.12) | 0.06 |
|  | G2-G3 | 126 | 0.16(0.11-0.22) | 61 | 0.16(0.10-0.30) | 0.76 |
|  | G4-G5 | 70 | 0.18(0.13-0.24) | 15 | 0.20(0.12-0.26) | 0.88 |
|  |  |  |  |  |  |  |
| Corticosterone(ug/l) | G1 | 591 | 1.44 (0.86-2.93) | 266 | 1.53(0.86-3.17) | 0.66 |
|  | G2-G3 | 133 | 1.19 (0.60-2.20) | 67 | 1.15(0.76-3.04) | 0.26 |
|  | G4-G5 | 73 | 1.71(0.33-3.20) | 18 | 1.33(0.84-3.22) | 0.91 |
|  |  |  |  |  |  |  |
| 17OH-progesterone  (ng/ml) | **G1** | **591** | **0.25 (0.16-0.41)** | **266** | **0.37(0.26-0.52)** | **<0.0001** |
|  | G2-G3 | 133 | 0.52 (0.38-0.74) | 67 | 0.54(0.39-0.80) | 0.78 |
|  | G4-G5 | 73 | 0.73 (0.56-1.03) | 18 | 0.66(0.59-0.87) | 0.59 |
|  |  |  |  |  |  |  |
| DHEA (ug/l) | **G1** | **591** | **0.89 (0.41-1.73)** | **266** | **1.95 (1.07-2.90)** | **<0.0001** |
|  | G2-G3 | 133 | 3.12 (2.32-4.17) | 67 | 3.14 (2.15-5.18) | 0.37 |
|  | G4-G5 | 73 | 4.10 (2.88-5.48) | 18 | 4.13 (3.07-6.05) | 0.96 |
|  |  |  |  |  |  |  |
| Androstenedione (ug/l) | **G1** | **591** | **0.18 (0.11-0.29)** | **266** | **0.29 (0.21-0.40)** | **<0.0001** |
|  | G2-G3 | 133 | 0.53 (0.37-0.67) | 67 | 0.57 (0.38-0.69) | 0.82 |
|  | G4-G5 | 73 | 0.71 (0.57-0.87) | 18 | 0.66 (0.50-0.81) | 0.35 |
|  |  |  |  |  |  |  |
| FT (pg/ml) | **G1** | **591** | **1.14(0.70-1.67)** | **266** | **1.64(1.09-2.49)** | **<0.0001** |
|  | **G2-G3** | **133** | **17.37**  **(8.83-27.75)** | **67** | **10.67**  **(5.29-19.44)** | **<0.0001** |
|  | G4-G5 | 73 | 34.08  (24.68-45.70) | 18 | 28.74  (23.75-39.00) | 0.26 |
|  |  |  |  |  |  |  |
| SHBG (nmol/L) | **G1** | **170** | **106.95**  **(79.68-148.45)** | **63** | **57.4**  **(40.10-84.90)** | **<0.0001** |
|  | G2-G5 | 93 | 34.90  (27.50-45.10) | 18 | 34.50  (17.75-41.30) | 0.11 |

Values are presented as median and interquartile range (IQR). NW: normal weight. OW/OB: overweight/obese. FT: Free testosterone. DHEA: Dehydroepiandrosterone. SHBG: Sex hormone-binding globulin.
